# Use of biomass-derived adsorbents for the removal of petroleum pollutants from water: a mini-review

**DOI:** 10.1186/s40068-021-00229-1

**Published:** 2021-04-07

**Authors:** Azar Vahabisani, Chunjiang An

**Affiliations:** Department of Building, Civil and Environmental Engineering, Concordia University, Montreal, QC H3G 1M8 Canada

**Keywords:** Petroleum pollutants, Adsorption, Biomass, Water

## Abstract

Over the past decades, a large amount of petroleum pollutants has been released into the environment resulting from various activities related to petrochemicals. The discharge of wastewater with petrochemicals can pose considerable risk of harm to the human health and the environment. The use of adsorbents has received much consideration across the environmental field as an effective approach for organic pollutant removal. There is a particular interest in the use of biomass adsorbent as a promising environmentally-friendly and low-cost option for removing pollutants. In this article, we present a review of biomass-derived adsorbents for the removal of petroleum pollutants from water. The features of different adsorbents such as algae, fungi, and bacteria biomasses are summarized, as is the process of removing oil and PAHs using biomass-derived adsorbents. Finally, recommendations for future study are proposed.

## Background

Over the past decades, a large amount of petroleum pollutants has been released into the environment resulting from various activities related to petrochemicals (Liu et al. 2020; Pi et al. 2019). The released petroleum pollutants in water have garnered increasing attention within the research community (Tian et al. 2020; Zhu et al. 2020). Hydrocarbon pollutants from contaminated wastewater which are discharged into the environment may have negative effects on marine life (Chen et al. 2020). The discharge of wastewater with petrochemicals can pose considerable risk of harm to the human health and the environment. The endangerment of various species has led to an elevated awareness and sense of urgency with respect to pollution control and the pressing need for countermeasures to address these issues.

The main methods for the treatment of wastewater with petroleum pollutants are physical, biological, chemical, and physicochemical treatment, dissolved air floatation, and mechanical separation (An et al. 2017). The use of adsorbents has received much consideration across the environmental field as an effective approach for organic pollutant removal (Cai et al. 2019; Shen et al. 2017). Biosorption, as a subset of adsorption, refers to the physicochemical adsorption and ion exchange occurring on the cellular surface of organisms. Because this phenomenon does not involve metabolism, it occurs in all cells, whether living or dead. Biosorption occurs through the binding to materials derived from various biomasses. It appears clear that using inactive and dead cells could be advantageous in the removal of pollutants, since toxic pollutants may have no effect on such cells, which are, therefore, relatively easy to handle. Furthermore, dead cells do not need any further treatment or nutrition and can be deployed in applications of this nature over many cycles. The use of algal biomass as a biosorption matrix is also a promising environmentally-friendly and low-cost option for removing the dissolved fractions of petroleum pollutants (Hubbe et al. 2014). In this regard, Aksu and Kutsal (1990) conducted research which shows the biomass of *Chlorella Vulgaris* has the potential to adsorb pollutants, including heavy metals ions, to the same extent as, or perhaps to a greater extent than, living cells. In a similar vein, Tam et al. (2002) investigated the removal of tributyltin through the adsorption on dead microalgal cells.

Some recent studies have proposed the use of biosorption to eliminate crude oil and polycyclic aromatic hydrocarbons (PAHs) from contaminated environments (Christensen and Rorrer 2009; Olivella et al. 2013). Various types of biomass, such as bacteria, fungi, algae, and plant cuticles, have been investigated as potential means to eliminate pesticides, dyes, heavy metals, and organic pollutants (Aksu and Tezer 2005; Chung et al. 2007; Wu and Yu 2006). Moreover, these studies have showed that using biomass could be an effective, reliable, and economical means of removing pollutants from aquatic environments (Zhang et al. 2013). One advantage of using such biosorbents is that the cost associated with the removal of pollutants is lower than that incurred by ion exchange, while it provides comparable removal performance for various pollutants (Davis et al. 2003). In this article, we present a review of biomass-derived adsorbents for the removal of petroleum pollutants from water. The features of different adsorbents such as algae, fungi, and bacteria biomasses are summarized, as is the process of removing oil and PAHs using biomass-derived adsorbents. Finally, recommendations for future study are proposed.

## Characteristics of biomass

With respect to biosorption, the use of different types of live and dead biomass, including algal biomass, fungi, and bacteria, has been reported (Carolin et al. 2017). Some important features of biomass make it an ideal option for removal, for example that it can be used on a large scale and is readily available. An ideal biosorbent will also be non-toxic and have high binding and regeneration/re-usability capacities (Wang and Chen 2009). The adsorption properties of biosorbents, it should be noted, are dependent on their structural characteristics, for example pore distribution, specific surface area, and functional groups (Ramrakhiani et al. 2011). However, some seasonal, temporal, and spatial environmental conditions may affect the chemical composition of biomass, such as temperature, ionic strength, pH, and natural light and nutrient availability (Peña-Rodríguez et al. 2011). Biomass can be well characterized for understanding the mechanisms about the binding of pollutants on biomass surface. It is important to characterize the structure and chemical characteristics of the cell surface for adsorption and separation processes. Different techniques may be deployed for this purpose, such as Fourier Transform InfraRed (FTIR) spectroscopy, X-ray Photo Electron Spectroscopy (XPS), Scanning Electron Microscopy (SEM), X-ray Diffraction (XRD), Energy Dispersive X-ray (EDX) fluorescence spectrophotometry, and surface area analysis. To obtain a comprehensive characterization of various biosorbents, these methods are commonly used together.

**Fig. 1:**
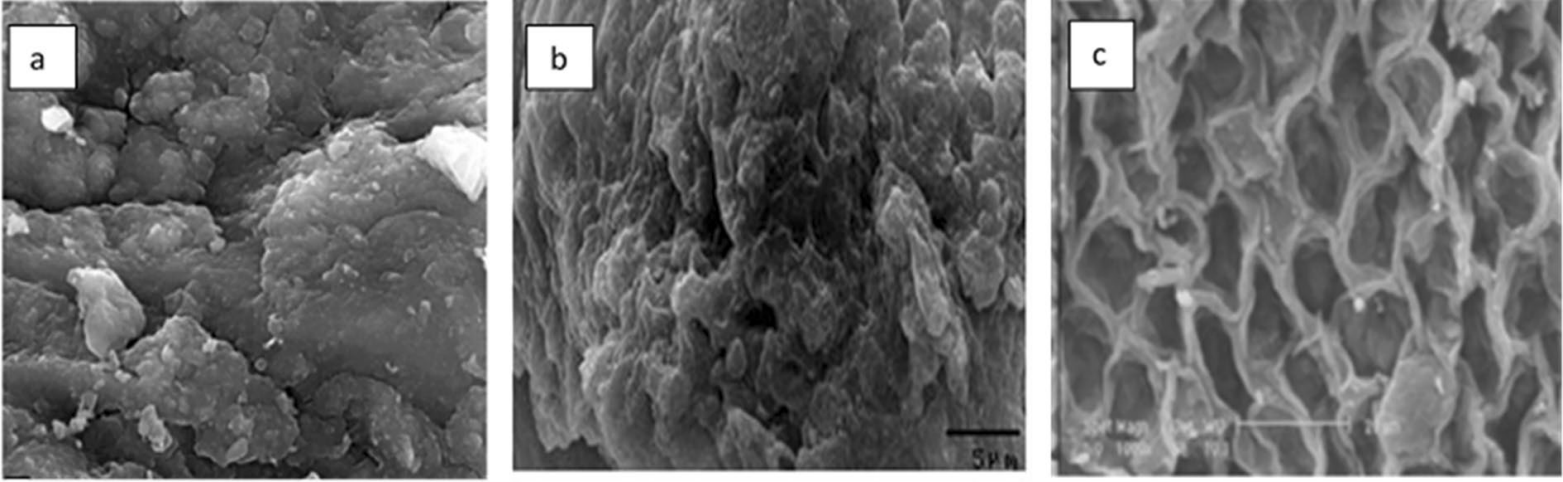
SEM images of **a** alga *Ulva lactuca* (Ibrahim et al. 2016)*, ***b** dried *Chlorella vulgaris* (El-Sheekh et al. 2019), **c** *E. intestinalis* biomass (Boleydei et al. 2018)
(ii) Fungi, bacteria and shells
  The structure of fungal cell walls contain chitin, a kind of polysaccharide which makes the cell structure rigid and strong. Fungal cell walls also contain glucans, which serve functions including ion exchange, rigidity, and metabolism. Polysaccharides account for 80% to 90% of the fungal cell wall. Two layers have been detected in ultrastructural studies of fungal cell walls (Arief et al. 2008). Bacteria are a type of unicellular organisms, existing widely in soil and water and in symbiosis with other organisms. Bacteria’s structure is relatively simple, lacking nuclei but possessing cell walls (Seltmann and Holst 2013). The bacteria have unique cell walls due to the presence of peptidoglycan. Since the peptidoglycan is porous, it has also been considered a barrier to small substrates.
  Rae et al. (2009) conducted research in regard to removal of Hg from acidic solutions with adsorbents derived from crab shell and found it to be effective, rapid, and easily processed. Cai et al. (2019), meanwhile, found the microstructure of crab shell powder to be characterized by a fibrous structure and a loose, unevenly arranged surface. Chitin is a natural polysaccharide consisting of (1–4)‐2‐acetamido‐2‐deoxy‐D‐glucose units, while chitosan is its deacetylated derivative. There are various sources that could provide chitin, such as fungi, insects, shrimps, and lobsters; however, the exoskeletons of crabs are the main commercial source (No et al. 1989). The amine and acetamido groups of chitin and chitosan can function as nonspecific binding sites, which are favorable for their use as biosorbents for removing pollutants. Furthermore, they are highly available and categorized as a low-cost sorbent. With regard to crab shell composition, Lee et al. (2004) showed that wastewater containing lead may be treated effectively with the presence of crab shell as it has chitin and calcium carbonate in its structure, and chitin could adsorb the lead- precipitant which calcium carbonate forms during treatment. Figure 2 shows the surfaces of the heat-dried fungus *A. malaysianum* biomass (Majumder et al. 2017), dried flakes of the bacterial cellulose (*Gluconacetobacter sucrofermentans*) (Atykyan et al. 2020), and crab shell powder (Cai et al. 2019).

(i) Algae
  Algae refer to the group of unicellular and multicellular organisms distributed in the aqueous environment which contain chlorophyl and have the ability to photosynthesise (Vahabisani et al. 2021). The characteristics of a given algal functional group may affect the adsorption process (Pathak et al. 2018). Algal biomass has been widely employed as a biosorbent material. Figure 1 shows the SEM images of the alga *Ulva lactuca* (Ibrahim et al. 2016), of dried *Chlorella vulgaris* (El-Sheekh et al. 2019), and of *E. intestinalis* biomass (Boleydei et al. 2018). Algae are considered an inexpensive and affordable biosorbent since no further treatment is needed. The characteristics of the algae cell wall make it a suitable biosorbent for heavy metals (Anastopoulos and Kyzas 2015). Non-living algal biomass may have the higher sorption capacity compare with living algae and nutrients are not required for such non-living algae (Zeraatkar et al. 2016). Different types of algae, such as marine red and brown macroalgae and freshwater green macroalgae, can be effective biosorbents. Some chemical compositions, such as alginate, which were reported in brown algae structure, give them a fair biosorption capacity (Bilal et al. 2018).
  The algae cell surface consists of various functional groups, such as carboxyl and sulphate groups, and also have components such as protein, lipid, and polysaccharides in their structure (Sarı and Tuzen 2008). These, in turn, are likely to have some bearing on the nature of the adsorption process (Henriques et al. 2017). Since non-living algal biomass can be effective in a toxic environment and does not need any media to grow, it has been used in various industrial applications to remove hazardous organic and inorganic pollutants. Carbohydrates are the main structural component of algae biomasses, and total carbohydrate sources vary according to species (Hernández-García et al. 2019). In a study conducted by Flores-Chaparro et al. (2017), carbohydrates were reported to account for 15.9%, 16.0%, and 11.8% of brown, green, and red macroalgae biomasses, respectively, followed by hemicellulose in the case of green (13.57%) and red (9.30%) macroalgae samples. For brown macroalgae, meanwhile, 13.4% protein content has been reported, which is among the highest compounds in the cell structure, while green and red seaweed only account for only 7.1% and 2.8% of the dry protein weight. The structure of cell walls of algae consists of biopolymers, polysaccharides, proteoglycans, and other molecular components. Macroalgae biopolymers are complex and several interactions between pollutants and active sites may occur simultaneously.

**Fig. 2:**
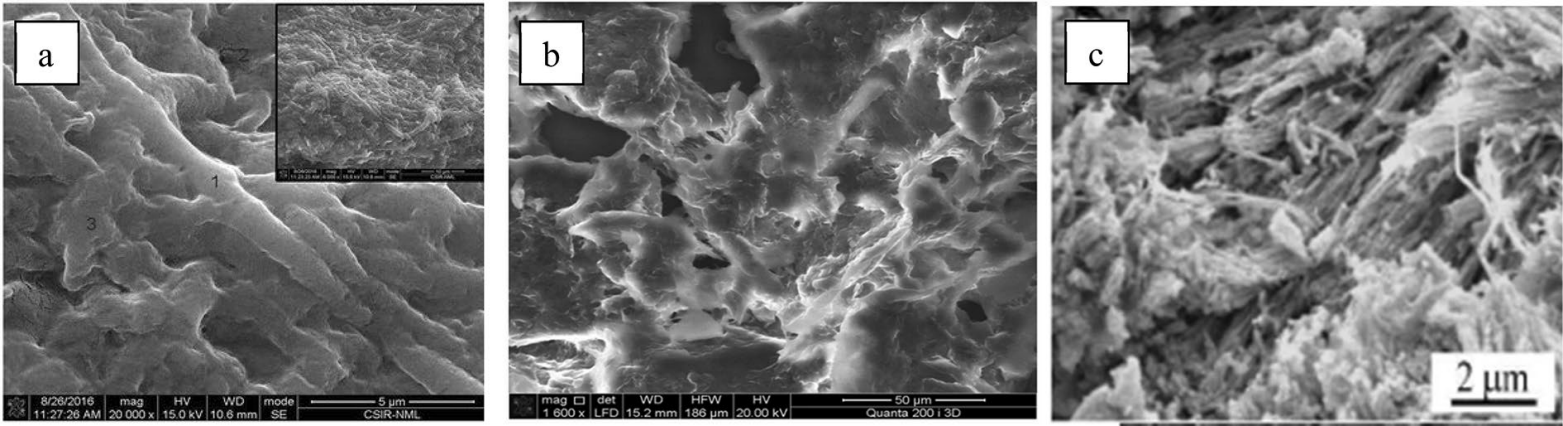
SEM images of **a** surface of heat dried fungus *A. malaysianum* biomass (Majumder et al. 2017), **b** dried flake of bacterial cellulose (*Gluconacetobacter sucrofermentans*) (Atykyan et al. 2020), (c) crab shell powder (Cai et al. 2019)

## Removal of petroleum pollutants from water using algae-derived adsorbents

### Removal of crude oil using algae-derived adsorbents

The removal of crude oil using algae-derived adsorbents was reported in some previous studies. Mishra and Mukherji (2012) studied the biosorption of diesel and lubricating oil on dead biomass of *Spirulina sp.* and *Scenedesmus abundans*. The sorption was studied in batch systems with different levels of oil and biomass. Based on their report, different biomass had different sorption rates, and *Spirulina sp.* could remove diesel in a short time. They analyzed the sorption rate and extent in batch systems containing 0.1% to 2% (v/v) oil and 0.1% biomass in water. They also found that both Freundlich and Langmuir models could fit for the sorption of diesel on algae but not for lubricating oil. A three-parameter model, meanwhile, was found to be suitable for all isotherms, which shows that maximum biosorption was in the range of 12 to 14 g/g for diesel and lubrication oil, respectively. In another study, Boleydei et al. (2018) reported the effectiveness of algal biomass of green macroalgae *E. intestinalis* as a biosorbent for decontamination of freshwater and seawater with crude oil and spent oil. Based on their results, sorption capacity for both crude oil and spent oil was higher in seawater than in freshwater. The spent oil, with a higher viscosity, showed higher sorption than the less viscous crude oil under the given conditions. Moreover, the adsorption data were found to be well fitted to the pseudo-second-order kinetic model and the Langmuir isotherm model.

**Fig. 3:**
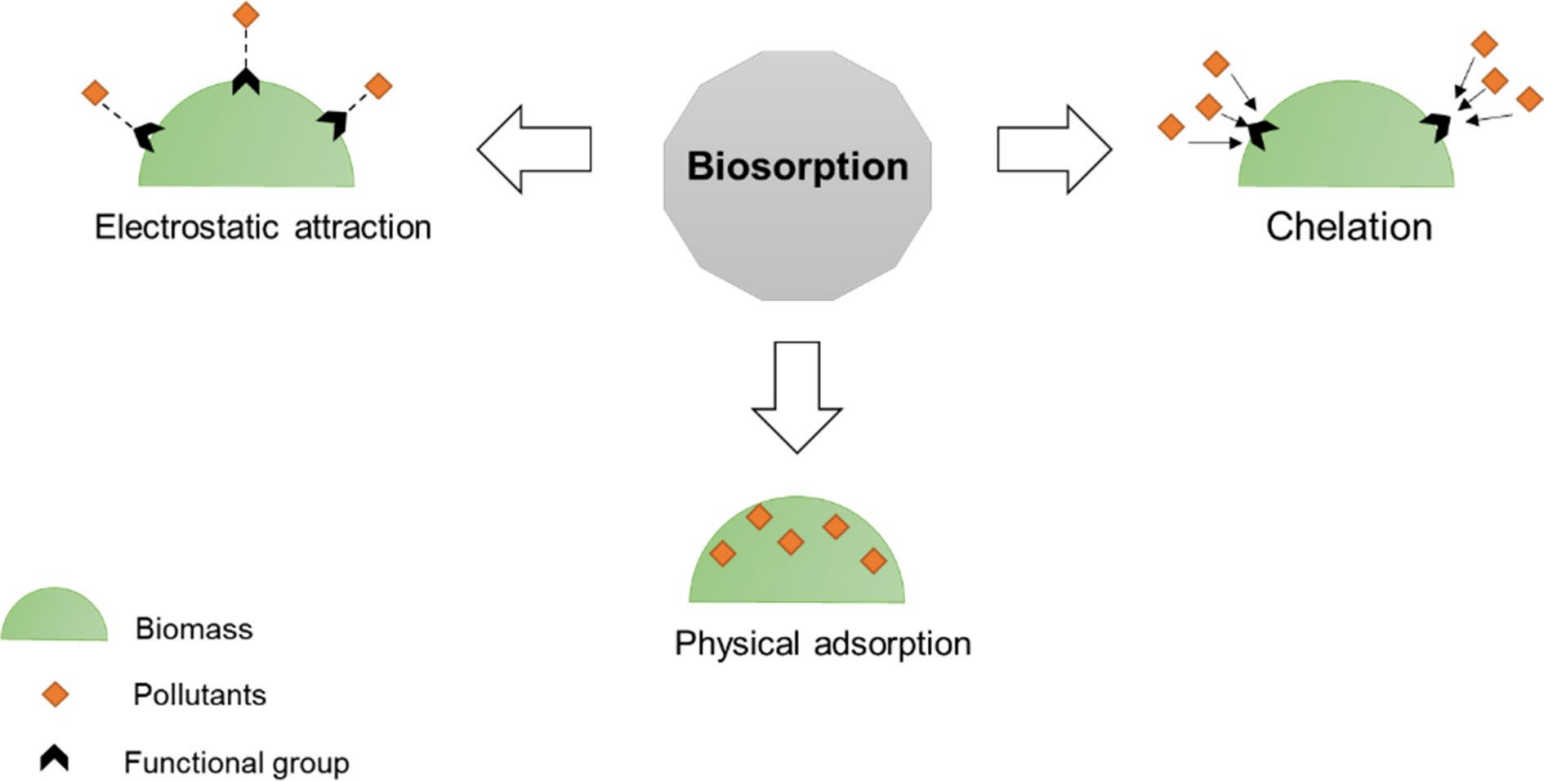
Adsorption, chelation/complexation and surface precipitation are different processes involved in biosorption

Different mechanisms have been proposed for the removal of toxic substances through biosorption (Bilal et al. 2018; Chojnacka 2010). Biosorption, as mentioned above, can occur as a passive process at a faster rate than bioaccumulation. Other processes, such as adsorption, chelation, and surface precipitation have all been identified as sub-processes of biosorption (Fig. 3). It is also noted that biomass type is related to biosorption mechanisms, with the particular mechanism of biosorption being dependent upon the biomass used for the removal (Flouty and Estephane 2012). As the cells were non-living, no metabolic energy was required (Avery et al. 1998). Various functional groups such as amino, carboxyl, sulphates, phosphates, and imidazoles, as well as the associated polysaccharides alginic acid and proteins on algal cell walls can bind with pollutants (Crist et al. 1981). In a natural environment, moreover, most algal cell walls have an overall negative charge, with receptors capable of attracting cations (Chu et al. 1997; Marbelia et al. 2016).

### Removal of PAHs using algae-derived adsorbents

PAHs naturally derive from fossil fuels, while they are also formed during the incomplete combustion of organic materials (Yu et al. 2011; Zhao et al. 2015). They have been found in the polluted water and soil. Studies have been conducted regarding the biosorption of PAHs on different microalgae (Pathak et al. 2018). Lei et al. (2007) presented the removal of PAHs, including fluoranthene (1.0 mg/L), pyrene (1.0 mg/L), and a mixture of fluoranthene and pyrene (each at 0.5 mg/L) by four microalgal biomass, *Chlorella vulgaris*, *Scenedesmus platydiscus*, *Scenedesmus quadricauda*, and *Selenastrum capricornutum*. Their study found the removal to be algal species-specific and toxicant-dependent. In their study, *Selenastrum capricornutum* was the species found to be most efficient at PAH removal/transformation (78%), whereas *C. vulgaris* was the least efficient in removing and transforming PAHs (48%). All the species under investigation, with the exception of *S. platydiscus*, showed better removal efficiency of fluoranthene than of pyrene. The removal rate of mixed fluoranthene and pyrene was comparable to or higher than the removal of either compound on its own, indicating that the presence of one PAH stimulates the removal of the other PAH.

The kinetics and equilibrium of the sorption of aqueous phenanthrene were studied by Chung et al. (2007), who used non-living biomass of the brown seaweed, *Sargassum hemiphyllum*, under different environmental conditions. Higher sorption of phenanthrene was observed with higher shaking speeds (50 to 250 rpm) and higher temperatures (15 to 35 °C), although there was no significant change with respect to maximum sorption capacities. Swackhamer and Skoglund (1993) have noted that hydrophobic substances are binding to the microalgal cell walls contents, such as cellulose and lipid, which affect biosorption process. Meanwhile, several studies have been conducted to evaluate the contributions of various fractions of algal debris on adsorptive removal of organic pollutants in order to better understand how such pollutants and debris interact. Non-living algal cells may release some substances from broken membranes to the medium, such as fatty acids and proteins under light (Kumar et al. 2019; Widrig et al. 1996). These findings suggest that some cellular contents can be released from broken membranes of dead algal cells and then induces the reactions with pollutants.

In a recent study, Zhang et al. (2019) presented the adsorption of three PAH compounds (phenanthrene, benzopyrene, and naphthalene) on green algae and reported that, after the removal of the lipid fractions from algae, the adsorption capacity decreased by as much as 25%. This indicates that the lipid fractions play an important role in the sorption of PAHs on algal biomass. The polysaccharide might have less effect on the adsorption of phenanthrene and benzopyrene, while the removal of polysaccharide had a positive effect on the adsorption capacity of naphthalene. Luo et al. (2014) hypothesized that PAHs may be highly adsorbed by non-living cells, which may be attributed to increased permeability of dead cell membrane in the absence of metabolic protection against the transport of pollutants into the cell, and changes to the surface adsorptive properties of the microalgae cell following its death. The adsorption is related to the property of algae detritus. The formation of more pores on the detritus after removing polysaccharides fraction could be favorable for the adsorption of pollutants.

Dead *Selenastrum capricornutum* has been shown to perform well at removing phenanthrene, fluoranthene, and pyrene (Chan et al. 2006). The PAH pollutant was removed primarily through a rapid adsorptive process, followed by plant uptake in the live algae. The principal mechanism involved could be physicochemical adsorption, which is metabolism-independent. On a related note, Avery et al. (1998) reported that organic and inorganic pollutants could bind to the cell surface of algal biomass due to the high potential of the binding sites. They also concluded that cell walls could provide many sites for potential binding of pollutants, and additional binding sites would be more readily available in dead cells than in live cells. Non-living cells have also been reported to have a higher capacity for PAH adsorption than live cells. Table 1 provides a summary of the various types of biosorbent used for petroleum pollutant removal.

**Table 1:** Use different types of algae-derived adsorbents in the removal of petroleum pollutants

Algae	Compound	Removal rate	References
*Spirulina sp. and Scenedesmus abundans*	Diesel and lubricant oil	Up to 75%	Mishra and Mukherji (2012)
*E intestinalis*	Crude oil and spent oil	80%	Boleydei et al. (2018)
*Sargassum hemiphyllum*	Phenanthrene	91.7–98.4%	Chung et al. (2007)
*Ulva prolifera*	Phenanthrene, benzopyrene, naphthalene	Not determined	Zhang et al. (2019)
*Chlamydomonas sp., Chlorella miniata, Chorella vulgaris, Scenedesmus platydiscus, S. quadricauda, S. capricornutum, Synechosystis sp.*	Pyrene, fluoranthene	Up to 78%	Lei et al. (2007)
*Selenastrum capricornutum*	Benz[a]anthracene, benzo[b]fluoranthene, benzo[k]fluoranthene, benzo[a]pyrene, dibenzo[a,h]anthracene, indeno[1,2,3-,d]pyrene, benzo[g,h,i]perylene	Not determined	Luo et al. (2014)
*Selenastrum capricornutum*	Phenanthrene, fluoranthene, pyrene	Up to 90%	Chan et al. (2006)
*Ulva prolifera*	Phenanthrene	91.3%	Zhang et al. (2017)

## Removal of petroleum pollutants from water using adsorbents derived from fungi, bacteria, and shells

### Removal of oil using adsorbents

**Fig. 4:**
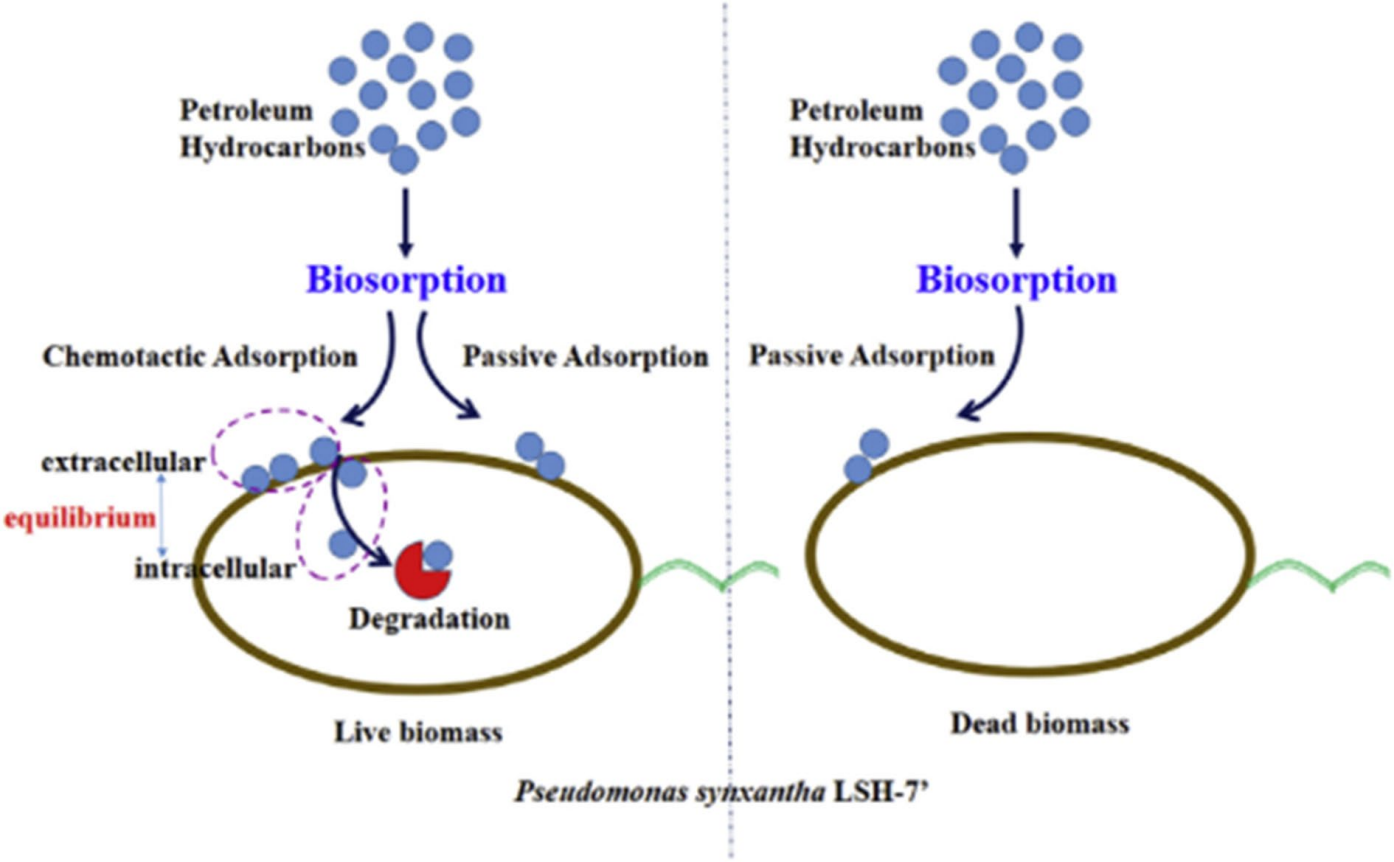
Sketch diagram of bacterial chemotactic biosorption (Meng et al. 2019)

The use of the dead cells of microorganisms such as fungi and bacteria as biosorbents has been studied since they are ubiquitous in aquatic environments (Cheng et al. 2020). Xu et al. (2013) conducted a study in which the crude oil adsorption on bacterial cells’ dead surface gradually decreased in a specific experimental time, likely due to the fact that some compounds in crude oil, such as aromatics and asphaltenes, were adsorbed. Some components of crude oil could be adsorbed rapidly by dead cells. Since there were some polar organic pollutants in oil and the reversible adsorption occurred, some adsorbed crude oil components were further released from the dead cells as time went on. In another study, Devi et al. (2012) concluded that the colloidal and suspended organic matter in vegetable oil mill effluent could be removed by natural chitosan derived from crab shell. The optimum pH solution of 4 was observed for organic pollutant removal. It was also found the minimal adsorbent dosage would be required for the treatment. In a study conducted by Cai et al. (2019), the adsorption kinetics of crab-shell-derived biochar with mesoporous structures was studied. The authors found that the biochar had a high capacity to adsorb diesel oil (about 93.9 mg/g). This is due to the high specific surface area (2441 m^2^/g), high pore volume (1.682 m^3^/g), and unique surface characteristics with functional groups such as hydroxyl (–OH–) and carboxyl (–COO–) (Fig. 4).

### Removal of PAHs using adsorbents

Some studies have been conducted to investigate the transport role in the surface adsorption of different hazardous organic pollutants by dead and live microbial consortia (Kim et al. 2020; Tsezos and Bell 1989). Kumar et al. (2008) reported that dead fungal biomasses possess a high capacity for biosorption of toxic pollutants such as heavy metals. Moreover, studies on the effect of fungal biomass on organic pollutant removal such as phenolic removal suggest that higher efficiency is achieved when non-living biomass is used (Rao and Viraraghavan 2002). The removal of PAHs using adsorbents derived from fungi, bacteria, and shells was also reported. Xu et al. (2013) explored the use of four different bacterial biomasses, including *Pseudomonas sp., Bacillus sp.*, *Ochrobactrum sp.*, and *Pseudomonas *sp. for adsorption of petroleum hydrocarbons. Adsorption of PAHs achieved by the heat-killed microbial consortium was found to be constant. Due to its high hydrophobicity, pyrene was found to be adsorbed more efficiently than phenanthrene and benzopyrene. The stability of the surface adsorption of naphthalene, phenanthrene, pyrene, and crude oil by the microbial biomass followed a decreasing order, as follows: naphthalene > phenanthrene = pyrene > crude oil. It was also noted that adsorption by bacteria biomass is a fast process.

Raghukumar et al. (2006) conducted a study in which a marine fungus named *NIOCC* #312 was used as a biosorbent for the removal of PAHs such as phenanthrene in a contaminated aquatic environment. They reported that phenanthrene could be rapidly adsorbed to the cell surface of the fungus, asserting that heat-killed fungal biomass could be used for the adsorption of PAHs from contaminated sites. Chen et al. (2010) showed the effectiveness of fungal biomass on the biosorption of PAHs in aqueous solutions. Pollutants could be adsorbed onto dead cells through surface sorption, carbon partitioning, and chemical reactions. They have attributed these phenomena to the biosorption behavior of microbial cells with respect to PAHs, this, in turn, being governed by the distributional effects of the sorption–desorption process.

### Environmental factors influencing adsorption using marine biomass-derived adsorbents

Pollutant removal can be impacted by different conditions (An et al. 2010; He et al. 2018). Some biomass characteristics such as biomass age, growth medium, and surface area can have effects on biosorption (Pathak et al. 2018). Natural environmental factors, such as pH, temperature, and salinity, also have significant effects on pollutant removal (Malik et al. 2002; Wang and Chen 2006).

(i) pH
  Biosorption on bacteria, algae, and fungi is often impacted by pH conditions. pH has an effect on the solution chemistry and competition of sorbate ions. It also influences the activity of the functional groups on biosorbents. Several mechanisms have been proposed for oil sorption on algal biomass surface, such as the absorption/partitioning of oil onto organic matter and adsorption onto the surface of algae due to specific interactions (Boleydei et al. 2018). Variations in pH affect the adsorption as the hydrogen ion itself is a tough competing adsorbate (Puranik et al. 1999). Highly alkaline conditions (pH of 10 or higher) have been shown to decrease the biosorption efficiency (Lim et al. 2016). Changes in the zeta potentials of oil and algae at different pH levels can also affect the oil removal process.
(ii) Temperature
  The effects on biosorption of another environmental parameter, temperature, have also been investigated in a number of studies. Temperature has an important effect on the biosorption process. The variation in temperature can result in the change of adsorbate kinetic energy and biosorbent physical structure. In a previous study, Mishra and Mukherji (2012) found increasing temperature had a positive effect on oil sorption on algal biomass. The viscosity of oil usually decreases at higher temperature. As temperature increases, there is enhanced movement of adsorbate molecules from the adsorbent. The increasing temperature may facilitate the pollutant desorption from biomass adsorbent. It should be noted that the porosity and total pore volume of the biomass adsorbent can also increase with increasing temperature. In addition, high temperature may result in the swelling internal structure of the adsorbents and the increased penetration of larger oil molecules.
(iii) Ionic strength
  Various ions exist in the aqueous environment (Bi et al. 2020). Chung et al. (2007) identified a correlation between sorption and ionic strength. In their study, they showed that increasing the salinity levels decreased the sorption of phenanthrene by brown seaweed *Sargassum hemiphyllum*. However, it should be noted that the type of biomass adsorbents has a notable effect on surface sorption. The trend in specific pollutant uptake variation with ionic strength, meanwhile, could be significantly affected by the type of biomass. Mishra and Mukherji (2012) noted that the trend in adsorption amount difference at different salinity levels was related to algal biomass type. They observed, for instance, that by decreasing ionic strength to 0.01 M, the adsorption of lubricating oil and diesel on *Spirulina sp* increased*.* When ionic strength increased to 0.1 M, the adsorption capacity was reduced; when it was increased to 0.5 M, adsorption capacity increased. Sorption capacity of diesel and lubricating oil using *S. abundans* continuously decreased when ionic strength further increased to 1 M. Adsorption of both lubricating oil and diesel on *Spirulina sp.* was highest at the lowest ionic strength (background electrolyte, 0.01 M NaNO_3_).

## Conclusions

The present study summarizes the biosorption of petroleum pollutants by various biomass-derived adsorbents. Factors such as pH, temperature, and ionic strength may have a major effect on the biosorption process. It was noted that much previous research has discussed the biosorption process of single petroleum compounds. Biosorption of other organic pollutants such as crude oil and mixed PAHs has many unexplored aspects. Since there is more than one compound in the nature and a mix of various hydrocarbons may exist, the investigation into the biosorption of mixed organic pollutants is required. Evaluating the removal of other hydrocarbons using biomass-derived adsorbents can also provide the required knowledge for remediation applications. The effects of complicated environmental conditions on the pollutant removal are relatively unknown. There are various components in the biomass-derived adsorbents. To better understand the biosorption and removal mechanisms, such as interaction between biomass components and adsorption process, further research is required. Biomass-derived adsorbents offer a low-cost and inexpensive approach for pollutant removal. In future studies, it is expected that better and more selective biosorbents will be found and biosorption mechanisms will be further identified.

## Data Availability

Not applicable.

## Abbreviations

EDS: Energy Dispersive X-ray
FTIR: Fourier Transform Infrared
PAHs: Polycyclic Aromatic Hydrocarbons
SEM: Scanning Electron Microscopy
XRD: X-ray Diffraction
XPS: X-ray Photo Electron Spectroscopy
